# Multidimension cultivation analysis by standard and omics methods for optimization of therapeutics production

**DOI:** 10.1186/1753-6561-7-S6-P5

**Published:** 2013-12-04

**Authors:** Julia Gettmann, Christina Timmermann, Jennifer Becker, Tobias Thüte, Oliver Rupp, Heino Büntemeyer, Anica Lohmeier, Alexander Goesmann, Thomas Noll

**Affiliations:** 1 Institute of Cell Culture Technology, Bielefeld University, 33615 Bielefeld, Germany; 2 Bioinformatics Resource Facility, Center for Biotechnology (CeBiTec), Bielefeld University, 33615 Bielefeld, Germany; 3 Center for Biotechnology (CeBiTec), Bielefeld University, 33615 Bielefeld, Germany

## Background

During the last decades Chinese Hamster Ovary (CHO) cells have been extensively used for research and biotechnological applications. About 40% of newly approved glycosylated protein pharmaceuticals are produced in CHO cells today [1]. Despite the increasing relevance of these cells for biopharmaceutical production little is known about effects of intracellular processes on productivity and product quality.

In the last years supplementation of serum-free media with insulin - more and more replaced by IGF-1 and its analogue LongR^3 ^- was utilized to enhance product titer and quality. To compare the intracellular effects of these two supplements an antibody producing CHO cell line was cultivated in batch mode using insulin, LongR^3 ^or no growth factor as reference. Subsequently, different omics-techniques were applied to analyze medium and cell samples.

## Materials and methods

CHO cells producing an antibody were cultured in chemically defined serum-free medium TC-BN.CHO (Teutocell AG) with addition of 6 mM glutamine. Three cultivations (37°C, pH 7.1, 40% DO, 120 rpm) were performed in 2l-bioreactor systems with supplementation of 10 mg/l insulin or 0.1 mg/l LongR^3^. The third culture was untreated and served as reference. Samples were taken every 24 h.

Viable cell density and cell viability were measured using Cedex (Roche). Glucose and lactate were determined via YSI 2300 STAT Plus™ Glucose & Lactate Analyzer (YSI Life Science). Quantitation of antibody production was determined using POROS^® ^A columns (Invitrogen). N-Glycan abundance was analyzed by HPAEC-PAD method [2].

For RNA samples 'Total RNA NucleoSpin Kit' (Macherey-Nagel) was used. Quality and quantity of RNA were determined using Nano Drop 1000 (Peqlab) and Bioanalyzer (Agilent).

An in-house developed customized cDNA microarray with 41,304 probes was applied for transcriptome analysis. RNA was labeled using Agilent LIQUA Kit, one-color. Processing of microarray data was performed in ArrayLims and EMMA2 [3]. Raw data were standardized using Feature Extractor (Agilent) and LOWESS normalization.

## Results

**Figure 1:**
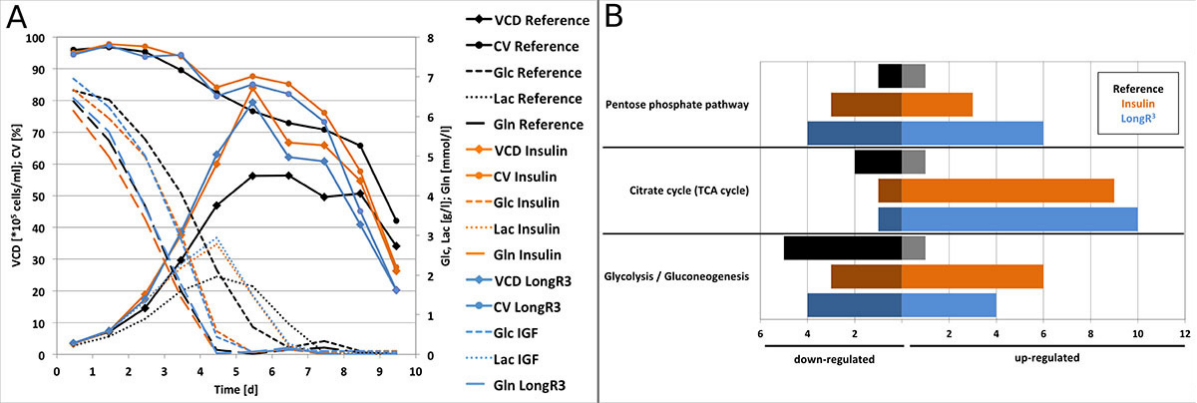
**(A)** Time chart of viable cell density (VCD), cell viability (CV) and extracellular metabolites [glucose (Glc), lactate (Lac), glutamine (Gln)]. **(B) **Number of significantly up- and down-regulated genes on day 5 in selected pathways (compared to day 3).

Cultivation data illustrated that maximal cell density was higher in cultivations with insulin and LongR^3 ^compared to that without growth factor. Additionally, glucose consumption and lactate production was slightly higher in cultivations with these supplements but time point of glutamine depletion was similar in all reactors after similar cultivation time (Figure 1A). Furthermore, product quantity and product quality was not influenced by growth factor addition. The most abundant glycoforms after 7 days of cultivation were G0F with about 50% and G1F with about 40% in all cultivation set-ups (Table 1).

**Table 1:** N-Glycan abundance [%] after 7 days of cultivation.

Culture	G0F	G0	G1F	G1	G2F	G2
**Reference**	51,8	4,1	35,6	0,9	7,4	0,2
**Insulin**	50,6	3,4	38,6	0,9	6,3	0,2
**LongR^3^**	52,5	1,4	38,5	0,5	6,9	0,3

For transcriptome analysis samples on day 5 were compared with those on day 3. Therefore, the following settings were used in statistical tests: a two-sample t-test with a p-value ≤ 0.01, signal intensity ≥ 6 (for A1 or A2) and intensity ratio ≥ 0.6 or ≤ -0.6 (for M1 or M2). Transcriptome data showed that LongR^3 ^supplementation resulted in the highest transcription change (1259 up- and 1689 down-regulated). Insulin supplementation resulted in second highest transcriptomic change (1026 up- and 1404 down-regulated) and reference cultivation led to lowest changes (344 up- and 301down-regulated). Supplemented cultures showed a higher transcription change in the selected pathways, like pentose phosphate pathway, TCA and glycolysis, than the reference culture, too. In LongR^3 ^containing cultures even more genes from these pathways were higher changed (Figure 1B).

## Conclusions

Data on cell growth and productivity as well as omics results were brought together to achieve a deeper insight into cellular processes and their influence on productivity and product quality.

Cultivation data showed faster growth, glucose consumption and lactate formation for cultivations with insulin and LongR^3 ^compared to reference culture. However, antibody titer and glycan profiles were almost similar in all cultures. This indicates that supplementation with insulin or LongR^3 ^does not have an enhancing effect on product quality and quantity in antibody production with our CHO-K1 cells.

Additionally, transcriptome data showed that growth factor supplementation resulted in a higher transcription change than in reference cultivation. Thus, for more understanding of the influence of insulin or LongR^3 ^supplementation on cultured CHO cells, further analysis of pathway regulation with full details is required.
